# Digital Technologies for Children and Parents Sharing Self-Management in Childhood Chronic or Long-Term Conditions: A Scoping Review

**DOI:** 10.3390/children8121203

**Published:** 2021-12-18

**Authors:** Jill Edwards, Jenny Waite-Jones, Toni Schwarz, Veronica Swallow

**Affiliations:** 1 School of Healthcare, University of Leeds, Leeds LS2 9JT, UK; j.m.waite-jones@leeds.ac.uk (J.W.-J.); v.swallow@shu.ac.uk (V.S.); 2 College of Health and Wellbeing and Life Sciences, Sheffield Hallam University, Sheffield S1 1WB, UK; t.schwarz@shu.ac.uk

**Keywords:** long-term conditions, chronic conditions, self-management, children, parents, mobile apps, digital technologies, scoping review

## Abstract

Worldwide, the prevalence of chronic (or long-term) conditions in children and young people from birth to 18 years (children) is increasing. Promoting competent and effective self-management skills early in the trajectory is important to improve adherence to treatment and optimise quality of life. Successful self-management, therefore, requires parents and children who are developmentally able to develop a range of complex skills, including the use of digital technologies. This scoping review aimed to identify primary research investigating digital technologies for children and parents sharing self-management in childhood chronic illnesses. A comprehensive search of electronic databases was conducted. Nineteen papers were included, assessed for quality and methodological rigour using the Hawker tool and thematically analysed. Three themes were identified: (i) the feasibility and acceptability of using technology, (ii) the usability of technologies and (iii) the effect of technologies on adherence and self-management skills. The results indicate that technologies such as mobile apps and websites can assist the management of long-term conditions, are an acceptable method of delivering information and can promote the development of effective self-management skills by parents and children. However, future technology design must include children and parents in all stages of development.

## 1. Introduction

Worldwide, the prevalence of chronic (or long-term) conditions in children and young people from birth to 18 years (children) is increasing [1]. A chronic condition is one that is incurable and requires daily medication and/or therapies [2]. Furthermore, the prevalence of multi-morbidities is rising for children, and patients with long-term conditions will in future absorb the largest and growing share of healthcare budgets [3,4]. 

There are strong arguments for promoting competent and effective self-management skills as early as possible in the disease trajectory as this is linked to good adherence to treatment regimens and consequently, optimal quality of life for affected individuals [5]. In addition, involving children in the design of interventions is most effective in improving self-management when interventions do not outweigh cognitive ability or maturity level [6]. However, self-management is challenging and often requires parents/carers (parents) and children to develop a range of complex skills and methods of managing the condition [7]. 

Modern healthcare systems are often reactive rather than proactive, but healthcare policies identify improved care for children with chronic conditions as a core aim, including the co-production with families of innovative tools for promoting effective condition management across the life course [1,8]. Internationally, paediatric services therefore need to move to a more proactive empowerment-orientated, family-focused approach to self-management of chronic conditions [9]. To meet this challenge, health services need to develop an approach that better coordinates and integrates support around the shared management requirements of parents and children living with chronic diseases [10]. Competent adherence to self-management during childhood is likely to enable optimum long-term outcomes in adulthood; this in turn will strengthen the cost-effectiveness of healthcare services [7,10]. Because chronic conditions increasingly involve children and parents in a considerable amount of skilled self-management, there is a growing need to consider and improve the self-management support available to them. [1,5,8].

The amount of self-management input required from parents and children depends on the range and type of home-based clinical interventions that are required and the child’s age, willingness and capability to gradually assume daily responsibility for managing their condition as they mature. Self-management frequently involves families in administering (parents) or self-administering (children) essential, complex and often unpleasant clinical procedures that can negatively impact adherence and quality of life [11]. The range of self-management skills may include, for example, frequent administration of unpleasant medications, delivery of supportive therapies, regular liaison with health care professionals, collecting prescriptions and medications, adjusting personal and family life to accommodate condition management and challenging and/or organising supportive services beyond the healthcare environment (e.g., applying for financial benefits or negotiating support at school or work). For clinical teams, it is also challenging to support these families to effectively undertake self-management procedures around-the-clock; this support can, however, be supplemented by reliable, evidence-based digital technologies [12]. 

In childhood chronic conditions, digital technologies have been found to support and empower patients and promote the development of a range of complex skills essential for effective management of the conditions, thereby promoting adherence to treatment regimens. A recent review noted that children may have important concerns and needs before using technology to self-manage their long-term condition [13]. Studies also suggest that interventions such as gamification (e.g., applying point scoring, competition, game juice and simulation to motivate people to achieve health-based goals) can improve child and parent quality of life [14,15]. Yet, condition management interventions are usually developed specifically for either children at specific ages or their parents but are rarely designed to be simultaneously relevant and accessible to both children and their parents. 

There is a need for good adherence to treatment regimens in chronic conditions, and digital technologies can significantly support this. However, there is also a need for rigorously developed and evaluated bespoke technologies to promote adherence by 5–18-year-olds and parents of children from birth to 18-years-old; such interventions need to take into account the child’s developmental trajectory and recognize that self-management is often actually shared management between the child and their parent(s) [16,17,18,19,20,21]. Yet, before intervention development, it is necessary to describe what is already known about self-management technologies for children and parents in chronic illnesses [22,23,24,25,26]. In summary, the evidence to date suggests a need for digital self-management interventions that enable and support shared management by parents of children from birth–18 years of age and children from the age of 5–18 years at different developmental stages across the life course. These interventions should be designed so that they can grow with children to address their individual needs at each developmental stage. Prior to intervention development, it is necessary to address this gap in our understanding; the aim of our scoping review therefore was: 

To identify primary research investigating existing digital interventions that address the needs of and enable and support shared chronic disease management by parents of children from birth–18 years of age and children from the age of 5–18 years to synthesise the findings and to determine whether further research is needed to develop and evaluate such interventions.

## 2. Materials and Methods

In conducting this scoping review, we followed the methodological framework provided by Arksey and O Malley (2005) [27]. We considered a scoping review to be the most relevant type of review for addressing our aims as it offers a broader and more flexible approach than a systematic type of review [27]. Scoping reviews are suited to identifying knowledge and research gaps prior to, for example, conducting a systematic review [28]. Scoping reviews increasingly assess the quality and methodological rigour of included studies e.g., [29,30] therefore, we used the Hawker Tool [31] because it is suitable for assessing the quality and rigour of studies with various designs and to indicate the level of confidence with which findings should be assessed. 

## 3. Eligibility Criteria

Once we had outlined our research aims, we developed the inclusion and exclusion criteria described below.

### 3.1. Inclusion

1. Children aged from 5–18 years with a long-term condition and parents of children from birth–18 years with a long-term condition.
2. Since a focus of this review was digital apps, we chose a publication start date of 2003. This is when the 3G networks arrived in the United Kingdom [24]. Mobile apps required this network to function.
3. All types of primary research studies that focused on using digital technology within self-management of physical long-term conditions by children and/or parents were considered for inclusion. We were interested in studies that explored how technologies may be used to develop self-management skills and knowledge to assist with self-management in our population group.

### 3.2. Exclusion

1. Studies not reporting primary research were excluded
2. Papers published prior to 2003 were excluded.
3. Papers that were not written in the English language as we did not have the resources to translate papers into English.
4. Studies that focused on children with cancer, mental health problems, learning disabilities and/or cognitive impairment were excluded due to resource limitations.
5. All literature reviews, editorials, letters, conference papers and PhD theses.

### 3.3. Identifying Information Resources

#### 3.3.1. Search Strategy

A search strategy was initially developed in Medline in conjunction with a university information scientist using a combination of standardised indexed search terms and free-text terms that related to key aspects of the research question; these were then adapted for use in other electronic databases to inform the final search strategy (see Appendix A).

To increase the sensitivity of the searches, abbreviations, variations on the spelling of key terms and synonyms were included. To identify sources related to medicine, psychology, behavioural sciences, nursing and allied health professions, a comprehensive search of CINAHL (Cumulative Index to Nursing and Allied Health Literature), Cochrane Database of Systematic Reviews, Cochrane Central Register of Controlled Trials, Science Expanded, Conference Proceedings Citation Index, Database of Abstracts of Reviews of Effect, Embase Classic + Embase (Ovid), International Clinical Trials Registry Platform (ICTRP), Ovid MEDLINE(R) (Medical Literature Analysis and Retrieval System Online, or MEDLARS Online), Ovid MEDLINEINPROCESS, PsycINFO (OVID), Assia (OVID) and Social Science Citation Index (Thomson Reuters Web of Science) was undertaken. To widen the results, in the advanced search engine, the Boolean operators “and” or “or” were used and adjacency of phrases was maintained by adding (adj) between words, e.g., “Long term adj conditions adj child”. Furthermore, using truncation (*, $) at the end of each keyword allowed retrieval of all possible results related to that word stem. The initial search was carried out in 2017 and updated in December 2019. 

**Figure 1:**
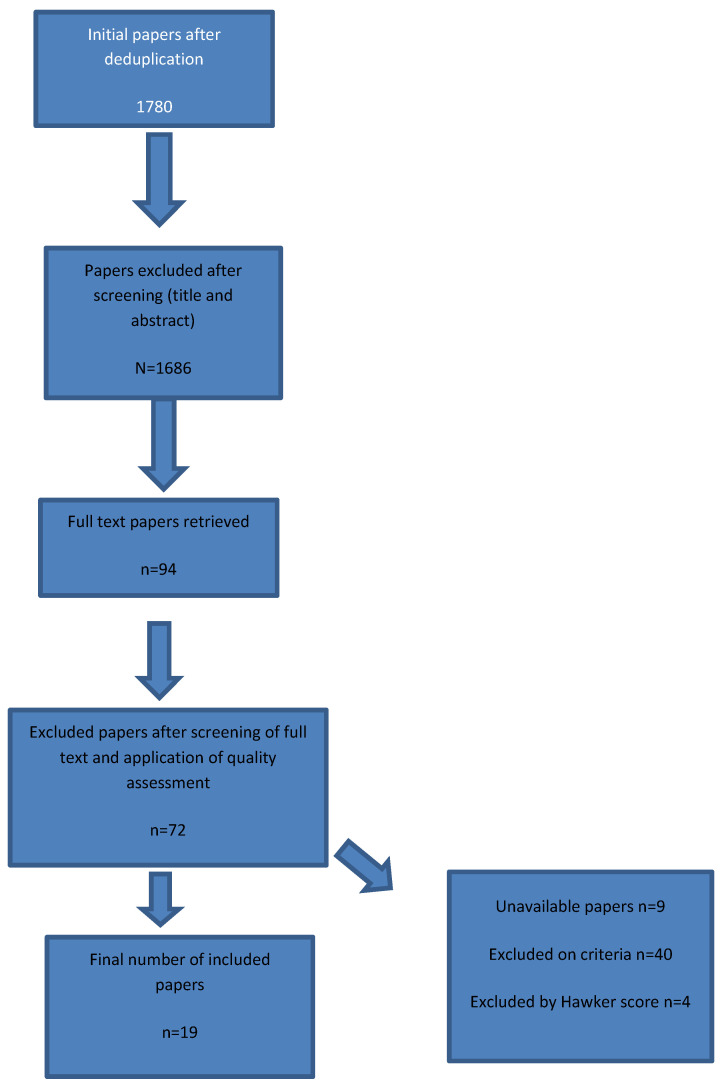
Prisma Diagram.

The total number of articles retrieved after de-duplication was 1780. Outlined below in the Prisma diagram (Figure 1) is the number of papers retrieved, included and excluded.

#### 3.3.2. Selection of Information Sources

The first reviewer (JE) screened all titles and abstracts against the inclusion/exclusion criteria in Thomas Reuters Endnote X9. A second reviewer (VS) then independently reviewed the titles and abstracts including those excluded by the first reviewer. Regular review meetings were held between the two reviewers to discuss and reach a final consensus on any differences of opinion which arose in relation to the inclusion or exclusion of papers. This approach has been applied in other scoping reviews [32]. Full-text articles of the included papers were then retrieved and screened, using the same approach. At this stage, all the authors worked in pairs to assess the full papers for inclusion using the standardised Hawker quality appraisal tool (2002) [31]. The Hawker tool is a method for reviewing qualitative, quantitative and mixed methods designs and disparate data systematically; it has a well-defined criterion and comprises nine items (1. Abstract and title, 2. Introduction and aims, 3. Method and data, 4. Sampling, 5. Data and analysis, 6. Ethics and bias, 7. Findings/results, 8. Transferability/generalizability and 9. Implications and usefulness) which papers are numerically sub-scored against. The sub-scores are then added up and each paper was given a final score which determines the overall quality of the paper (0–9 = very poor; 10–18 = poor; 19–27 = fair; 28–36 = good). Further clarification regarding calculating the summed score was previously sought and provided through email contact with one of the Hawker tool’s authors [7]. Our papers were either accepted or rejected based on their total score across the nine-item criteria. 

### 3.4. Quality Appraisal

A total of 72 full-text papers were critically appraised using the tool [31]. Papers were divided between the four authors for review, and each reviewer independently assessed their allocated papers for methodological validity and scored them against Hawker’s criteria. Papers that scored under 19 were excluded (*n* = 4). To check the level of concordance between reviewers, each reviewer independently appraised a sample of another reviewer’s papers, and scores were compared. While there was some variation in the scores, these did not differ substantially across criteria, and there was consistency in how the eligibility criteria were applied to the full-text papers. 

### 3.5. Data Extraction, Charting and Synthesis

A form was created for data extraction, where the reviewers extracted detailed information on each study. Arksey and O Malley (2005) [27] refer to this stage as ‘charting’ and it involves identifying key items of information from the included papers. The areas covered were: objectives, study design, participants, setting/context, aim/objectives, research questions/hypothesis (if any), methods and analysis, results and key conclusions. The information taken from each paper and entered on the data extraction form was then entered into excel spreadsheets in order to obtain an overview of all the included papers. 

Close examination of the charted information allowed us to determine if our initially chosen method of analysis (synthesis) of the papers could be applied across them all. At this stage, Arksey and O Malley [27] point out that important decisions must be made about how comparisons between different studies can be achieved. 

A thematic analysis was deemed relevant for the synthesis since it enables the inclusion of both qualitative and quantitative papers [33]. Our aim was to produce a descriptive narrative of the included studies. According to Arksey and O’Malley [27] (p. 27) scoping reviews do not attempt to present ‘a view regarding the weight of evidence in relation to particular interventions or policies. Munn et al. [28] (p. 18) state that the aim of a scoping review is not to produce ‘a critically appraised and synthesised result/answer to a particular question, but rather aim to provide an overview or map of the evidence’.

To identify themes, we familiarised ourselves with the key findings of each paper, using the charted data and referring back to the full papers when more detail or explanation was required. We focused on the key findings of each paper since the aim was to describe what is already known about self-management technologies for children and parents in chronic illnesses to identify gaps in our understanding of this area. The key findings of each paper were organised thematically, and using a ‘compare and contrast’ approach, we mapped the identified themes under headings which after further review were grouped together under three major themes. These are described below in the results section. 

## 4. Results

### 4.1. Summary of Papers

The final 19 papers reported studies that used a range of research designs: mixed methods, surveys, real-time assessments, feasibility studies, randomised clinical trials and an evaluation study. (Please refer to Appendix B ‘characteristics of papers’ for more details inserted at the end of this paper). The long-term conditions included were diabetes (*n* = 11), asthma (*n* = 4), pain (*n* = 2), arthritis (*n* = 1) and haemophilia (*n* = 1). Thus, there was a bias in the sample towards diabetes. Of the 19 studies included, 11 focused solely on children up to 18 years, seven included both children up to 18 years and parents/carers of children aged 0–18 years. One paper focused exclusively on fathers (which helped to address the issue of gender bias in the overall parent sample reported), although not all studies reported the gender of parental participants, so it was difficult to assess whether there was any substantial gender bias in our review. Most studies focused on older children and adolescents which potentially adds an age bias, and ethnic minorities were also likely under-presented, though few studies provided breakdowns of participants’ ethnicity except for St. George et al. [34], who focused on ethnic minority adolescents and their parents. 

Most of the studies examined the feasibility, usability or acceptability of using mobile apps, the internet or other forms of technology (such as video and text messaging) to deliver self-management programs or the support aspects of self-management (e.g., blood glucose monitoring) with the aim of improving knowledge, symptom awareness and adherence to treatments.

The findings from the review are presented below as a narrative summary within three key themes: (i) the feasibility and acceptability of using technology, (ii) the usability of technologies and (iii) the effect of technologies on adherence to treatment regimens and improving shared self-management skills. 

### 4.2. Theme 1: The Feasibility and Acceptability of Using Technology

Many of the papers focused on the feasibility and acceptability of using technologies such as mobile apps, text messaging and the internet to support self-management of chronic conditions [34,35,36,37,38,39,40,41,42,43,44,45,46]. 

The provision of information was regarded as essential to improving knowledge and symptom awareness across several conditions. Several studies examined the feasibility of providing self-management programs aimed at improving user knowledge of their condition via the internet [34,35,39,45,47].

Providing this information via internet websites and mobile apps was generally acceptable to adolescents and their parents and led to improvements in knowledge and symptom awareness [34,47]. Similarly, using a combination of a glucose meter and phone-assisted self-monitoring of glucose levels in adolescents with diabetes was acceptable. Text messaging and the use of mobile apps were found to improve symptom awareness triggers and promote adherence in adolescents with diabetes and asthma [34,36,37]. 

Using video conferencing facilities to deliver specialized behavioural health care was found to have potential advantages over traditional outpatient psychotherapy, including reducing travel time for patients and improving accessibility to speciality providers. Moreover, the growing accessibility of high-speed Internet services in rural communities has made the use of videoconferencing an increasingly achievable means of delivering behavioural health in these areas [48]. Furthermore, although limited time may be available for parental interventions in clinic settings, remote treatment programs have the potential to enable multiple family members to access skills training in the home and point to the potential value of offering low-intensity treatments such as cognitive behavioural therapy (CBT) to all parents of children with chronic pain [48].

While it was found that using these forms of technology generally improved self-management across different conditions [35,36,37,38,41,43,44,46], some negative outcomes were reported. Gammon et al. [38] found that the transfer of glucose results from adolescents’ phones to their parents had the potential to exacerbate pre-existing conflict in families. Concerns expressed highlighted the potential to create a negative feeling of surveillance and increased nagging and scolding by the parents; the outcome of this was for children to stop monitoring their blood glucose to avoid the exacerbation of conflicts [38].

### 4.3. Theme 2: The Usability of Technologies

Several papers addressed the issue of usability when using technologies to support self-management [34,45,46,47,49,50]. The importance of well-designed programs that involved users in all stages of the design process was highlighted as having the potential to influence user engagement with the final version of the technology. Stinson et al. [45] found that satisfaction with a web-based program was related to the aesthetics of the user interface, content, functionality and features and opportunities to receive social support from other users and professionals. Stinson et al. [45] also reported that it is important to design any program around user needs and preferences to promote usage of the intervention/s, while Padman et al. [50] identified game mechanics and social media features as key factors in engaging users in using mobile apps. 

Interest in using apps for monitoring glucose levels and/or symptoms and participating in internet programs varies across youths and parents [34]. The level of interest was found to be affected by users’ ability to and understanding of how to navigate the internet and mobile apps. Clements et al. [49] aimed to assess the impact of data synchronization between a glucometer and a mobile app, and they found that 42 per cent of participants failed to synchronize data to the app despite receiving training about the importance of doing this. The authors concluded that additional support and training may be needed to assist users or that additional motivators to drive synchronization behaviour may also be necessary [49]. 

The usability of web-based information and education programs appeared to be enhanced with access to a health professional or coach and interactive features [46,47]. Such provision appeared to promote motivation in areas such as self-monitoring of glucose levels in patients with diabetes. Moreover, it highlighted a need for participants to receive feedback and suggestions for problem-solving exercises. 

Differences in preference between users may need to be considered when designing web-based programs to ensure uptake and continued engagement. For example, Stinson et al. [45] found differences between parents and adolescents over the presentation of text. While parents did not mind reading large amounts of text, adolescents preferred that the text was broken up with visual assets. Similarly, Whittemore et al. [45] found that adolescents preferred instant methods of communication via text messaging and social media. 

### 4.4. Theme 3: The Effect of Technologies on Adherence to Treatment Regimens and Improving Shared Self-Management Skills 

Some studies focused on evaluating the effect of web-based programs and mobile apps on adherence to treatment regimens and improving self-management skills [39,41,51,52] Chan et al. [52] considered whether adherence and disease control were improved through using an internet-based store and a forward video telehealth system versus face-to-face consultations. Assessments via this method were found to be effective and acceptable in the short term to patients compared to the face-to-face option. However, over the long term, they found no significant difference between patients assigned to face-to-face consultations compared to the telehealth system. Levels of adherence may be affected by patients’ ability to cope with carrying out daily treatments. Grey et al. [39] compared two internet-based psycho-educational programs aimed at improving quality of life and HbA1c; one program focused on teaching a wide range of coping skills to adolescents and the other was designed to teach advanced diabetes problem solving and healthy lifestyles using an interactive format. While participation in each program separately yielded some improvements in outcome measures, the authors concluded that participation in both programs was more likely to produce better outcomes overall (suggesting that adolescents require both sets of skills to successfully transition to adolescence).

Borus et al. [51] found that social influences had the potential to impact whether patients aged 14–18 would monitor their blood glucose. They found that a desire to fit in with peers sometimes overwhelmed the need to check their glucose levels despite them knowing the consequences of not checking their blood glucose. In the study by Harris et al. [41], statistically significant improvements in adherence and glycemic control occurred from before to after the intervention, involving Skype to deliver Behavioural Family Systems Therapy for Diabetes compared to clinic visits, and improvements were maintained at a three-month follow-up [41].

## 5. Discussion

This scoping review has achieved our aim by identifying primary research investigating existing digital technologies for children and parents sharing self-management in childhood chronic illnesses and by synthesising the findings. The synthesis revealed three key themes to help inform future intervention development: (i) the feasibility and acceptability of using technology, (ii) the usability of technologies and (iii) the effect of technologies on adherence to treatment regimens and improving self-management skills. Our findings suggest a lack of evidence-based digital interventions (i) to assist parents of children from birth–18 years to share condition management with their children as appropriate and (ii) that are developmentally appropriate for children from the age of 5–18 years and that can grow with them as they mature. 

We believe this is the first scoping review to identify and synthesise primary research investigating self-management technologies for both children and parents sharing self-management to determine whether there is sufficient evidence for future intervention development in long-term conditions. 

This review highlights the importance of both child and parent involvement in the co-design of any interventions; this is more likely to ensure that interventions are responsive to the users’ needs and preferences and therefore feasible and accessible. This in turn will assist with adequate information provision and enable symptom awareness [39,45,47]. 

Although providing information via internet websites and mobile apps was generally acceptable to adolescents and their parents and led to improvements in knowledge and symptom awareness, there were examples of disparity between child and parent views on this. For example, Gammon et al. [38] found that the transfer of glucose results from adolescents’ phones to their parents had the potential to exacerbate pre-existing conflict in families. This finding emphasises the importance of collaborating with both children and parents as early as possible when co-producing self-management interventions and of listening to and, where possible, responding to their disparate views by producing intervention components that are specifically targeted to the needs of each group [53]. To optimise accessibility and feasibility, interventions need to include targeted, developmentally appropriate and regularly updated components for children of different ages and developmental stages.

Overall, the findings of this review suggest that providing information and other resources online should not replace regular face-to-face consultations. Having regular access to health advice appeared to encourage adherence to treatment, ensure continued motivation and provide clarity for individual patients and their parents. In other studies, access to information and online learning about their condition is considered helpful to supplement the information and advice given face-to-face by health professionals [20]. 

Our findings indicate several examples of the ways in which technologies are accessible, feasible and usable and positively impact adherence, but that several gaps remain. The most important gap we identified is a lack of disease-specific interventions designed to meet the shared management needs of both children (developmentally appropriate) and parents, although some generic aspects of existing interventions may be transferrable across conditions. There is, therefore, some merit in considering the non-categorical approach to chronic illness, which postulates that children face common life experiences and problems based on generic dimensions of their conditions and not just on idiosyncratic characteristics of their specific disease entity. The non-categorical approach argues for a generic rather than, or as well as, a condition-specific focus on dimensions that vary across disease categories rather than on disease-specific differences. Therefore, there is scope to consider any of the issues arising from our review that may be relevant across conditions [54]. 

Text messaging and the use of mobile apps were found to improve symptom awareness triggers and promote adherence in adolescents with diabetes and asthma, so it is fair to assume that these media could also be feasible and acceptable for children with other conditions [36,37,44]. The lives of children and their families are affected by whether the chronic condition is visible or invisible, whether it is life-threatening or stable and the effect on children of repeated hospitalizations and days lost from school can be examined regardless of the condition that led to hospitalization. Yet, a condition-specific approach also merits consideration as indicated in recent qualitative studies [20,55] that sought children’s, parents’ and professionals’ views on desirable components for a future digital care management app in chronic kidney disease and chronic rheumatic disease. Reported gaps in current online information and support were related to content, trustworthiness, child-friendliness and accessibility. Children, professionals and parents of older children thought the information on websites was quite basic, lacked detail and was unhelpful in developing self-confidence in self-managing the condition [20]. Furthermore, healthcare support apps should be interactive and engaging, offering a sense of ownership and control, and they should provide developmentally appropriate information, monitor symptoms, offer reminders and enable access to social support. All participants concluded that online information could be “scary,” “dry,” “boring” and was either hard to understand or not relevant to individual circumstances. There was concern that websites may not contain accurate information and, therefore, may not be reliable, useable or accessible; moreover, for some children, searching online was a much less accessible approach than using a mobile app.

## 6. Strengths and Limitations

The review aimed to represent geographical diversity, although most studies included were conducted in the USA. Therefore, the results have potential application internationally. When interpreting the review results, some methodological limitations also need to be considered. For example, our focus on papers written in the English language potentially limits their transferability to contexts where English is not widely spoken. The existence of the digital divide (the gap between individuals, households, businesses and geographic areas at different socio-economic levels with regard to both their opportunities to access information and communication technologies (ICTs) and to their use of the Internet for a wide variety of activities) may limit some families’ access to online resources [56]. There were insufficient data in the selected papers to report on how age, income, ethnicity, geographic location and disability may have impacted families use of technologies. However, it is already known that all these factors impact access to the internet [57]. For example, a combination of lack of skills to use the internet and levels of income determine how likely it is that a family or individual will have internet access [57]. The presence of a child with a disability and/or with a long-term condition can impact income; it is reported that 48% of people living in poverty, totalling 6.8 million people in the UK, live in a family where someone is disabled [57]. However, there were few RCTs or comparative effectiveness trials to answer questions regarding in-person vs. virtual counselling vs. online/digital tools or generic chronic disease vs. specific disease management approaches. 

## 7. Future Research

This review identified and synthesised the existing evidence and revealed a need for rigorously developed, evidence-based, user-led and developmentally appropriate digital self-management technologies for children aged 5–18 years with long-term conditions and parents of children from birth–18 years who need to share self-management for optimum benefit. Future research needs to focus on developing interactive information and support resources that are evidence-based, co-produced with families and professionals and based on their preferences and suggestions. Future research, in particular using mixed methods and RCTs, is also needed to determine how age, income, ethnicity, geographic location and specific long-term conditions may be supported by digital technologies. These issues would need to be assessed during the development of any digital technologies. 

The findings of this review will inform international healthcare practitioners about the feasibility, acceptability and usability of technologies in their practice to (i) support children with chronic conditions as they learn about self and shared management and (ii) about the potential effects of technologies on adherence to treatment regimens and improving self-management skills. This review revealed several areas where healthcare providers can assist children and parents by co-producing and directing families to rigorously developed and evaluated, user-led self-management interventions designed to empower individuals within families as they undertake shared management. Child healthcare providers can also help families by informing them about and supporting their use of relevant, evidence-based digital interventions to support self and shared management of long-term conditions. Before recommending specific interventions, however, practitioners should also consider the digital divide and the potential for some families being unable to access digital technologies to aid self and shared management.

## 8. Conclusions

This review identified and synthesised primary research investigating digital technologies for children and parents sharing self-management in childhood chronic illnesses. Three themes were identified: (i) the feasibility and acceptability of using technology, (ii) the usability of technologies and (iii) the effect of technologies on adherence and self-management skills. These findings highlight the importance of both child and parent involvement in the co-design of any interventions as this is more likely to ensure that the interventions respond to users’ identified needs and preferences and are therefore feasible and accessible. This in turn will assist with adequate information provision and enable symptom awareness. Future research needs to focus on developing interactive, developmentally appropriate information and support resources that are evidence-based, co-produced with families and professionals and based on their preferences and suggestions.

## Appendix A. Search Strategy

	**Query**	**Limiters/Expanders**
S45	S7 AND S30 AND S41 AND S44	Search modes—Boolean/Phrase
S44	S42 OR S43	Search modes—Boolean/Phrase
S43	(MH “Self Care+”)	Search modes—Boolean/Phrase
S42	TI “self help” or “self care” or “self manage *”	Search modes—Boolean/Phrase
S41	S31 OR S32 OR S33 OR S34 OR S35 OR S36 OR S37 OR S38 OR S39 OR S40	Search modes—Boolean/Phrase
S40	TX iPhone * or “mobile health” or digital or video * or game * or quiz * or blog * or twitter * or tweet * or Instagram or “social media”	Search modes—Boolean/Phrase
S39	TX (smartphone * or phone * or ipad *)	Search modes—Boolean/Phrase
S38	(MH “Smartphone+”)	Search modes—Boolean/Phrase
S37	(MH “Computer Assisted Instruction”)	Search modes—Boolean/Phrase
S36	TI (online or telemecidine)	Search modes—Boolean/Phrase
S35	TX (mobile N2 device *)	Search modes—Boolean/Phrase
S34	TI ((cell * or mobile *) N3 phone *)	Search modes—Boolean/Phrase
S33	(MH “Social Media”)	Search modes—Boolean/Phrase
S32	(MH “Computers, Hand-Held+”)	Search modes—Boolean/Phrase
S31	(MH “Cellular Phone+”)	Search modes—Boolean/Phrase
S30	S8 OR S9 OR S10 OR S11 OR S12 OR S13 OR S14 OR S15 OR S16 OR S17 OR S18 OR S19 OR S20 OR S21 OR S22 OR S23 OR S24 OR S25 OR S26 OR S27 OR S28 OR S29	Search modes—Boolean/Phrase
S29	(MH “Chronic Disease”)	Search modes—Boolean/Phrase
S28	TX long term condition *	Search modes—Boolean/Phrase
S27	TI ((long-term or life-long or “long term” or “life long” or progressive or degenerative or non communicable or non-communicable) N2 (“health problem *” or illness * or condition * or disease * or disorder *))	Search modes—Boolean/Phrase
S26	TI (chronic * N1 (problem or illness * or condition * or disease * or disorder *))	Search modes—Boolean/Phrase
S25	TI ((chronic * or “long term” or long-term or life-long or “life long”) N1 pain)	Search modes—Boolean/Phrase
S24	(MM “Chronic Pain”)	Search modes—Boolean/Phrase
S23	(MM “Pain”)	Search modes—Boolean/Phrase
S22	TX asthma *	Search modes—Boolean/Phrase
S21	TX (epilepsy * or epileptic *)	Search modes—Boolean/Phrase
S20	TX ((brain N2 (“cancer * or malignanc *” or neoplasm *or tumor *))	Search modes—Boolean/Phrase
S19	TX sarcoma *	Search modes—Boolean/Phrase
S18	TX (haematologic * N2 (“cancer * or malignanc *” or neoplasm * or tumor *))	Search modes—Boolean/Phrase
S17	TX (hematologic * N2 (“cancer * or malignanc *” or neoplasm * or tumor *))	Search modes—Boolean/Phrase
S16	TX diabetes	Search modes—Boolean/Phrase
S15	(MH “Status Asthmaticus”)	Search modes—Boolean/Phrase
S14	(MH “Asthma, Exercise-Induced”)	Search modes—Boolean/Phrase
S13	(MH “Asthma+”)	Search modes—Boolean/Phrase
S12	(MH “Epilepsy+”)	Search modes—Boolean/Phrase
S11	(MH “Neoplasms+”)	Search modes—Boolean/Phrase
S10	(MH “Sarcoma+”)	Search modes—Boolean/Phrase
S9	(MH “Hematologic Neoplasms+”)	Search modes—Boolean/Phrase
S8	(MH “Diabetes Mellitus+”)	Search modes—Boolean/Phrase
S7	S1 OR S2 OR S3 OR S4 OR S5 OR S6	Search modes—Boolean/Phrase
S6	TX infant or infancy or infants or “young people” or “young person” or “young adult *” or “schoolchild *” or neonat * or preterm * or prematurity or postmature * or baby or babies or toddler *	Search modes—Boolean/Phrase
S5	(MH “Child, Preschool”)	Search modes—Boolean/Phrase
S4	(MH “Young Adult”)	Search modes—Boolean/Phrase
S3	(MH “Pediatrics+”)	Search modes—Boolean/Phrase
S2	(MH “Infant+”)	Search modes—Boolean/Phrase
S1	TX (child or children or childhood or adolescen * or teen or teens or teenager * or youth or youths or girl or girls or boy or boys or *p* * ediatric * or juvenil *)	Search modes—Boolean/Phrase

## Appendix B. Characteristics of Papers

	**Authors**	**Title of Paper**	**Population**	**Intervention**	**Control**	**Outcome**	**Design of Study**	**Hawker Tool Quality Assessment Total Score**	**Country**
1	Albanese-O’Neil, A, Schatz D.A, Bernhardt JM & Elder, JH. 2016	Educational needs and technological preferences of Fathers of youth with type 1 Diabetes	Fathers of adolescents 6–17 years.	Identified the educational needs and technological preferences of fathers of youths.	No	Data supported feasibility/acceptability of diabetes education via mobile technology. Found interest in receiving education via smartphone technology. High levels of unmet needs in diabetes education.	mixed methods	31	USA
2	Borus JS, Blood E, Volkening LK, Laffel L & Shrier LA. 2013	Momentary assessment of social context and glucose monitoring adherence with type 1 diabetes	36 participants aged 14–18 years. Mean age 16.6.	Explored the association between sociocontextual factors and glucose level monitoring.	No	Identified some social influences on monitoring blood glucose. Found desire of individuals to blend in with friends may support glucose monitoring and desire to impress others may impede it.	Real time assessments	24	USA
3	Breakey VR, Ignas DM, Warias AV, White M, Blanchette VS & Stinson J. 2014	A pilot randomized control trial to evaluate the feasibility of an internet-based self-management and transitional care program for youth with hemophilia	29 participants. 13 –18 years.	Evaluate internet based self-management and transitional care program for hemophilia.	Yes	Significant improvements in knowledge in intervention group compared to control group. Web site found to be comprehensive and easy to use. Coach and weekly calls helped tailor learning to individual.	Feasibility (RCT)	30	Canada
4	Burbank AJ, Lewis SD, Hewes M, Schellhase DE, Rettiganti M, Hall-Barrow J, Bylander LA, Brown RH & Perry TT. 2015	Mobile-based asthma action plans for adolescents	20 participants. 13–18 years.	Examined feasibility and utilization of a mobile asthma action plan.	No	Utilization of mobile app by adolescents to deliver asthma action plan feasible. Asthma better controlled when app utilized.	Single-arm, feasibility and proof-of-concept study	22	USA
5	Carroll AE, Dimeglio LA, Stein S & Marrero DG. 2011	Using a cell phone-based glucose monitoring system for adolescent diabetes management	40 participants (36 completed study). 13–18 years. 19 girls and 20 boys.	Assess the feasibility of the combined phone and glucose meter system and the extent to which it would be acceptable to adolescents and parents.	No	The integrated phone and meter reported to be very useful and 75% of participates stated they would switch if available. Well-received by parents and adolescents. Using device increased knowledge and self-management.	Surveys	22	USA
6	Chan D, Callahan C, Sheets S, Moreno C & Malone F. 2003.	An Internet-based store-and-forward video home telehealth system for improving asthma outcomes in children	10 participants 6–17 years. 5 boys and 5 girls.	Test feasibility and effectiveness of internet-based education programme versus office-based education.	No	Adherence and disease control. Compared office face to face consultations with video home tele system. Some connectivity issues reported due to technical problems in rural areas. Time-consuming method for health professionals.	Randomised study	24	USA
7	Clements MA & Staggs VS. 2017.	A Mobile App for Synchronizing Glucometer Data: Impact on Adherence and Glycemic Control Among Youths with Type 1 Diabetes in Routine Care	81 participants 10–15 years Median age 14. 49% male	Whether mobile apps can improve self-management of glucose levels. Assessed impact of the frequency of glucometer-based data synchronization via a mobile app on HbA1c.	No	Found no statistical significance between rate of glucometer data synchronization and glucose control. Correlation to frequency of synchronization and monitoring of blood glucose.	Retrospective study/quantitative	24	USA
8	Gammon D, Arsand E, Walseth OA, Andersson N, Jenssen M, & Taylor T. 2005.	Parent-child interaction using a mobile and wireless system for blood glucose monitoring	30 participants children and adolescents (aged 9–15 years. Boys *n* = 11 Girls *n* = 4) and their parent. Mothers 9 Fathers 1 15 families in total.	Testing of prototype designed to automatically transfer readings from child’s glucose meter to parents’ mobile phone.	No	Found self-management improvements of blood glucose levels and quality of life. Potential to increase pre-existing conflict between parents and children. Children concerned about increased parental surveillance. May be biased to those who are confident with technology	Pilot/mixed methods	22	Norway
9	Grey M, Whittemore R, Jeon S, Murphy K, Faulkner M & Delamater A. 2013	Internet psycho-education programs to improve outcomes with type 1 diabetes	320 participants 11–14 years. 55% female.	Comparison of two internet programmes on primary outcomes HbA1c and quality of life (QoL).	No	Adherence/QOL- compared two internet programmes to teach coping skills. QOL improved and HbA1c improved. Low income may affect engagement due to limited access to internet.	Clinical trial	27	USA
10	Han y, Faulker M, Fadoju D, Muir A, Abowd G, Head L & Arriaga R. 2015.	A Pilot Randomized Trial of Text-Messaging for Symptom Awareness and Diabetes Knowledge in Adolescents with Type 1 Diabetes	30 participants aged 10–17 years (27 completed the study). Mean age 13.7%, 57% female	Piloting of text messages and examination of effects on symptom awareness and knowledge of diabetes.	Yes	Found some improvements in quality of life for adolescents in symptom awareness and knowledge (SK) group. No significant changes in QOL at baseline and follow up in control group and symptom (S) group. Diabetes quality of life for youth (DQOLY)- satisfaction did increase for S and SK groups.	Mixed methods	22	USA
11	Harris M, Freeman K & Duke D. 2015	Seeing Is Believing: using Skype to Improve Diabetes Outcomes in Youth	90 participants 12–18 years and their carers. Mean age of participants 15.04 and 55% male. Most carer mothers 76.7%.	Effectiveness of delivery of online behavioural family systems therapy (BFST) & glucose monitoring. Viable via video conferencing to address non-adherence.	No	Improvements found before and after the intervention and were maintained at 3 month follow up. BFST effective via video conferencing and Conventional method (i.e., in clinic setting).	Quantitative	25	USA
12	Hosseini A, Buonocore CM, Hashemzadeh S, Hojaul H, Kalantarian H, Sideris C, Bui AAT, King CE & Sarrafzadeh M.2017.	Feasibility of a Secure Wireless Sensing Smartwatch Application for the Self-Management of Pediatric Asthma	2 participants. 1 child (7–12 years) and 1 adult	Assess whether a child could wear and use smartwatch app to alert to potential asthma attacks.	No	During feasibility testing, system able to accurately assess asthma risk in adult and during usability testing it was able to continuously collect data and alert to potential asthma attacks. Found child could wear smartwatch and understand alerts.	Feasibility	22	USA
13	Padman R, Jaladi S, Kim S, Kumar S, Orbeta P, Rudolph K & Tran T. 2013.	An evaluation framework and a pilot study of a mobile platform for diabetes self-management: insights from pediatric users	8 participants. 10–18 years.	Evaluation of app for diabetes self-management	No	Found positive trends in user engagement and blood glucose variability. Increased satisfaction with diabetes management. Detected high variability in user’s interaction with application.	Evaluation	23	USA
14	Palermo T, Law E, Fales J, Bromberg M, Jessen-Fiddick T & Tai G. 2016.	Internet-delivered cognitive-behavioural treatment for adolescents with chronic pain and their parents: a randomized controlled multicentre trial	273 adolescents aged 11–17 years and their parents	Comparison of internet delivered cognitive behavioural therapy and internet education.	Yes	Effectiveness of CBT treatment via internet. Reduction in depression, anxiety and self-blame.	Quantitative	36	USA
15	Palermo T, Wilson A, Peters M, Lewandowski A & Somhegyi H. 2009.	Randomized controlled trial of an Internet—delivered family cognitive-behavioral therapy intervention for children and adolescents with chronic pain	48 participants children and adolescents aged 11–17 years & parents	Explored acceptability of internet CBT programme for children and parents.	Yes	Significantly greater reduction in activity limitations and pain intensity at post treatment for internet group and effects maintained after 3 months. Internet treatment acceptable to children and parents.	Quantitative	35	USA
16	Rhee H, Allen J, Mammen J & Swift M. 2014.	Mobile phone-based asthma self-management aid for adolescents (mASMAA): a feasibility study	15 participants-dyads 9 males and 6 female 13–17 years 12 mothers	Development of self-management aid for adolescent with asthmas using texting feature. Feasibility and acceptability of mobile app for asthma self-management via text messaging.	No	Improved awareness of symptoms and five parents believed system useful and beneficial for those whose asthmas was not controlled well. General acceptable and feasible to use the app. Some difficulties in initial connection to system.	Qualitative	36	USA
17	St George SM, Delemater SM, Pulgaron ER, Daigre A & Sanchez J. 2016.	Access to and Interest in Using Smartphone Technology for the Management of Type 1 Diabetes in Ethnic Minority Adolescents and Their Parents	Participants 50 adolescents aged 11–16 (52% female) and 49 parents	Examined access and interest in using web based and smartphone technology for managing diabetes in ethnic minority adolescents.	No	Most participants reported high interest in using diabetes specific app. More parents than adolescents expressed interest in using it. Identified barriers to use but majority had access to smartphones and internet.	Quantitative	32	USA
18	Stinson J, McGrath P, Hodnett E, Feldman B, Duffy C, Huber A, Gtucker L, Hetherington R, Tse S, Spiegel L, Campillo S, Gill N & White M.2010.	Usability Testing of an Online Self-management Program for Adolescents with Juvenile Idiopathic Arthritis	38 participants aged 12–18 years and parents.	Explored the usability of the self-management program for youth and parents.	No	Found adolescents more comfortable using computers than parents. Interactive features of program made them feel supported and not alone in coping with their illness.	Qualitative	36	Canada
19	Whittemore R, Grey M, Lindemann E, Ambrosino J & Jaser S. 2010.	Development of an Internet coping skills training program for teenagers with type 1 diabetes	12 participants aged 13–16 years. Mean age 14.4 58% female.	Development of an internet coping skills training program	Yes	Found feasible and acceptance of internet coping skills training programme. Developing online resources complex and requires multi-disciplinary approach. Wide range of skills needed for coping, and email not good way to communicate with teens.	Mixed methods	25	USA
